# Handbook of Diseases of the Eye

**Published:** 1885-03

**Authors:** 


*Handbook of Diseases of the Eye.* By H. R. SWANZY. Pp. 437. London: H. K. LEWIS. 1884.

The book before us would seem particularly well suited for the use of busy practitioners. It contains full information on Diseases of the Eye; it is well arranged, pleasantly written, and printed in good type.

Symptoms, causes, and prognosis are adequately discussed; treatment is very clearly and thoroughly dealt with. Conjunctivitis, a disease of much importance in family practice, is treated at unusual length. The physician will find full information on affections of the optic nerve and retina. There is a good practical chapter on sympathetic ophthalmitis, and glaucoma receives the space that its importance demands; it is carefully considered with all recent knowledge on the subject. Operations are well illustrated, and fully described, especially those for cataract and plastic procedures on the lids.

The chapters dealing with the dioptric apparatus of the eye, and errors in refraction, are, we think, less satisfactory. Nothing is more important in the treatment of asthenopia than a thorough knowledge of the use of spectacles; indeed scarcely any case comes under the care of the ophthalmic surgeon where some help from lenses is not given, and the physical, physiological, and therapeutic aspects of hypermetropia and short-sight cannot be too thoroughly dealt with, and might be most satisfactorily combined in a student's handbook. The methods of experimenting on the acuteness of vision, and on the condition of the visual field, are not up to the general standard of the book; whilst retinoscopy and color blindness are briefly but very ably described. Perhaps the best work in the book is that on the action and impairment of the pupil and of the orbital muscles.

Mr. Swanzy's book will be found most reliable and useful to those of us who, in the busy exercise of all branches of the art of healing, may require aid in dealing with diseases of the eye. Its author has every right to speak with authority.

